# Intention to Use Pre-Exposure Prophylaxis Among Age Groups of Brazilian Men Who Have Sex With Men: National Cross-Sectional Study

**DOI:** 10.2196/58405

**Published:** 2025-07-08

**Authors:** Alvaro Francisco Lopes de Sousa, Caique Jordan Nunes Ribeiro, Guilherme Reis de Santana Santos, Emerson Lucas Silva Camargo, Herica Emilia Félix de Carvalho, Guilherme Schneider, Leticia Genova Vieira, Liliane Moretti Carneiro, Ana Paula Morais Fernandes, Talita Morais Fernandes, Márcio Bezerra-Santos, Rita de Cassia Dias Nascimento, Lucas Almeida Andrade, Anderson Reis de Sousa, Inês Fronteira, Isabel Amélia Costa Mendes

**Affiliations:** 1 Public Health Research Centre, Comprehensive Health Research Center, CHRC, REAL, CCAL, NOVA National School of Public Health, Universidade Nova de Lisboa, Avenida Padre Cruz, Lisbon, 1600-433, Portugal, 351 21 751 2100; 2 Instituto de Ensino e Pesquisa, Hospital Sírio-Libanês, São Paulo, Brazil; 3 ››Graduate Program in Nursing, Universidade Federal de Sergipe, São Cristovão, Brazil; 4 Ribeirão Preto College of Nursing, Universidade de São Paulo, RIbeirão Preto, Brazil; 5 Universidade Estadual do Maranhão, Campus Coroatá, Maranhão, Brazil; 6 Program in Health and Development in the Central-West Region, Universidade Federal de Mato Grosso do Sul, Campo Grande, Brazil; 7 Ribeirão Preto Faculty of Medicine, Universidade de São Paulo, Ribeirão Preto, Brazil; 8 Medical and Nursing Science Complex, Universidade Federal de Alagoas, Arapiraca, Brazil; 9 Nursing College, Universidade Federal da Bahia, Salvador, Brazil; 10 Graduate Program in Health Sciences, Universidade Federal de Sergipe, Aracaju, Brazil

**Keywords:** age groups, pre-exposure prophylaxis, HIV, men's health, sexual and gender minorities, PrEP, men who have sex with men, MSM, sexual behaviors, preventive practices, Brazil, HIV prevention, epidemic, global trends, social stigma, cross-sectional study, electronic survey, prevalence, syphilis, infections, sexually transmitted disease

## Abstract

**Background:**

In Brazil, men who have sex with men (MSM) are disproportionately affected by the HIV epidemic, mirroring global trends. Despite advancements in HIV prevention, such as pre-exposure prophylaxis (PrEP), uptake remains uneven among different MSM age groups, influenced by various sexual behaviors, risk perceptions, and social stigmas.

**Objective:**

This study aimed to investigate factors associated with PrEP intentions among Brazilian MSM across different age groups.

**Methods:**

A cross-sectional electronic survey was conducted with Brazilian MSM aged 18 years and above.

**Results:**

PrEP intentions were expressed by 55.1% (2390/4341) of participants. Among those under 25 years old, frequent barebacking was associated with a 25.4-fold higher prevalence of PrEP intentions (95% CI 18.18‐35.48). Conversely, having one or more casual sexual partners in the last 30 days (2164/3838) was associated with a 22% lower frequency of PrEP intentions (95% CI 0.72‐0.85). The practice of chemsex was also associated with a lower prevalence of PrEP intentions (adjusted prevalence ratio [aPR] 0.96; 95% CI 0.94‐0.98). Among individuals aged 25‐49 years, the practice of double penetration was associated with a higher prevalence of PrEP intentions (aPR 1.22; 95% CI .13‐1.32), as was being a receptive or versatile partner (aPR 1.27; 95% CI 1.04‐1.55 and aPR 1.23; 95% CI 1.01‐1.49). In addition, within this age range, a higher prevalence of PrEP intentions (over 10% [258/429]) was found among those with specific characteristics, such as a previous diagnosis of syphilis (aPR 1.12; 95% CI 1.03‐1.22). However, being single (aPR 0.90; 95% CI 0.83‐0.97) and having a partner who used PrEP (aPR 0.82; 95% CI 0.72‐0.93) were associated with a lower prevalence of PrEP intentions. For MSM aged ≥50 years, a higher prevalence of PrEP intentions was associated with the practice of double penetration (aPR 1.31; 95% CI 1.20‐1.43), as well as being a receptive (aPR 1.27; 95% CI 1.04‐1.55) or versatile partner (aPR 1.23; 95% CI 1.01‐1.50). The practice of group sex was independently associated with a lower prevalence of PrEP intentions exclusively among MSM aged ≥50 years (aPR 0.82; 95% CI 0.75‐0.89).

**Conclusions:**

The study highlights significant generational differences in factors influencing PrEP intentions among Brazilian MSM. It underscores the need for tailored HIV prevention strategies that consider the unique behaviors and perceptions of different age groups. By addressing these nuances, public health initiatives can more effectively promote PrEP use, catering to the diverse needs of the MSM community and contributing to the reduction of HIV infection rates.

## Introduction

Men who have sex with men (MSM) experience a disproportionately high burden of HIV infection compared to the general population and even other groups vulnerable to the virus [1-4]. Since the beginning of the HIV epidemic in the 1980s, MSM have been recognized as one of the most affected populations due to factors such as social stigma, discrimination, barriers to accessing health care services, and higher-risk sexual behaviors. These longstanding challenges have made HIV a significant public health concern among MSM, despite advances in infection prevention and treatment [3,5].

According to the World Health Organization (WHO), in 2022, approximately 39 million people were living with HIV worldwide, with 29.8 million receiving antiretroviral therapy, contributing to a 62% reduction in the AIDS mortality rate since 2000 [6]. However, treatment does not fully meet the needs of the MSM population, highlighting the need to target Antiretroviral Therapy across different age groups to reduce new HIV infections [7].

The number of individuals infected with HIV varies based on age and sexual behavior. In Brazil, approximately 1 million people live with HIV, with a growing proportion of infections among those aged 13 years to 39 years, particularly among MSM (526/1.000, 52.6%). However, the prevalence within this group is not uniform and varies by age group. According to official Brazilian reports, the HIV prevalence among younger MSM (aged 13‐24 y) is 24% (240/1.000), among young adults (aged 25‐34 y) it is 40% (400/1.000), and among those over 35 years old, it stands at 17% (170/1.000). These rates are significantly higher than those observed in the general Brazilian population within the corresponding age groups, at 0.3%, 0.7%, and 0.8%, respectively [8].

The differences in HIV/AIDS prevalence among MSM of various age groups can be attributed to several factors. Understanding how sexual behaviors vary with age is a critical aspect of epidemiology in this population, as it significantly impacts HIV exposure risk [9-13].

Older MSM are more likely to exhibit risk factors for HIV infection, which may include, in addition to cumulative exposure over time, increased stigma and discrimination that can impede access to healthcare services and HIV-related information, including testing, antiretroviral treatment, and condom use [14-17]. This population continues to represent a substantial portion of those living with HIV. According to WHO data, in 2022, 22% of people living with HIV worldwide were MSM, with 17% of them aged 35 years or older [6]. In Brazil, the proportion of HIV diagnoses among individuals aged 50 years and older increased from 9.3% to 12.7% between 2009 and 2019 and continues to rise each year [16].

On the other hand, young MSM are more prone to engaging in risk behaviors due to new forms of relationships and sexual practices that increase their vulnerability to HIV acquisition and other sexually transmitted infections (STIs). One example is chemsex, the practice of using drugs to enhance sexual experiences, which has gained popularity among younger MSM. Chemsex is associated with prolonged sexual encounters, reduced inhibition, and less consistent condom use, all of which contribute to higher risks of HIV transmission [18-20]. In addition, younger MSM are more likely to engage in casual relationships, often facilitated by dating apps, leading to multiple sexual partners and further increasing their likelihood of HIV exposure [21,22].

HIV prevention efforts, such as pre-exposure prophylaxis (PrEP), often focus predominantly on young MSM, while there is limited literature on the subject regarding older MSM [15,23]. PrEP has been shown to be effective in preventing HIV among MSM of all ages. For instance, a study conducted in the United States demonstrated that PrEP is equally effective among young MSM (aged 18‐25 y) and older MSM (aged 26‐50 y) [24]. Consequently, HIV prevention efforts, including PrEP, should be expanded to encompass older age groups as well.

The objective of this study was to investigate factors associated with PrEP intention among Brazilian MSM of different age groups. We hypothesized that there are differences in the factors associated with PrEP intention among MSM of different age groups residing in Brazil.

## Methods

### Study Design

This is a cross-sectional online survey study that includes MSM throughout Brazil, aged 18 years and older, from January 2020 to May 2021.

### Population and Sample

A sample size calculation was performed using G*Power software (version 3.1.9.7; Heinrich Heine University Düsseldorf) to determine the sample size needed for the study. The calculation considered the male population in Brazil, a tolerable SE of 3% , a confidence level of 95%, and a presumed outcome prevalence of 50%.

It is noteworthy that when it is not possible to determine the true prevalence of the phenomenon of interest in local or national literature, a presumed prevalence of 50% is used, as this value maximizes the sample and its use does not pose a problem [25,26].

Participants who identified as men (cis or trans), had sexual relations with another man in the last 12 months, and were 18 years or older were included.

Individuals diagnosed with HIV infection and those already using PrEP were excluded from the study.

### Data Collection Procedures

To recruit survey participants, we used the snowball sampling technique adapted for the virtual environment, a method previously validated for the MSM population [9,27]. In this approach, participants help recruit other individuals with similar characteristics through their social networks and contacts by sending them an invitation to participate. Given that our study was conducted in a country of continental dimensions, we intentionally selected the first 30 participants with diverse social and demographic characteristics—including age group, region or district of residence, race or skin color (White or non-White), income, and education—to enhance the generalizability of our data [9,27,28].

To reach these initial 30 participants without requiring researchers to travel across all regions of Brazil, 2 of our cisgender male researchers, who identify as MSM and were thoroughly trained and calibrated with one another, created public profiles on 2 geolocation-based dating apps, Grindr and Hornet, to identify the “seeds.”

The decision to use Grindr and Hornet was based on their widespread recognition as popular platforms within the MSM community, offering a broad user base. In addition, their geolocation features facilitated a more targeted and efficient approach to identifying participants in various regions of Brazil. Another significant factor was the familiarity and comfort many MSM community members already have with these platforms, as they are commonly used for social interaction and relationship-seeking.

Participants were offered the option to maintain anonymity until they felt comfortable disclosing their identity to the researchers, fostering a safe and welcoming environment for study participation.

This innovative strategy not only optimized the recruitment process but also demonstrated our commitment to inclusion and cultural sensitivity, which are crucial for the success of the research.

Within these apps, researchers approached the first available online individuals who met the study’s inclusion criteria and satisfied the criteria for seed generalization, as recommended by previous studies [29,30]. After engaging with each seed, we provided a link to participate in the study and instructed them to invite other MSM from their social networks until reaching the required sample size through a dissemination strategy.

### Data Collection Instruments

The survey form was hosted on the SurveyMonkey platform for data collection. This platform incorporates security features that permit only one response per IP address. To ensure the validity of the survey form, a content and face validation process was conducted with 10 expert judges in the field. In addition, a pretest involving 10 participants was carried out to assess the clarity and effectiveness of the form.

The study’s dependent variable was the intention to use PrEP, with outcomes categorized as yes or no. The explanatory variables included sociodemographic data such as age, education level, place of residence, gender identity, and relationship status. Participants were also queried on their practices related to disclosing HIV status on dating apps, their knowledge of PrEP, and the “undetectable=untransmittable” concept, as well as their previous diagnoses of STIs and history of HIV testing, either in their lifetime or within the past 12 months.

Subsequent sections of the instrument examined variables associated with sexual practices, including preventive measures taken during sex to avoid HIV transmission, sexual activity with a partner living with HIV, involvement in group sex (defined as sexual encounters involving 3 or more people), self-assessment of perceived HIV infection risk, preferred sexual positions, involvement in receptive anal sex without a condom, recent engagement in unprotected sex, drug use during sexual encounters or sex under the influence of drugs within the last 6 months, sexual activity in public places, and specific practices such as bareback sex, double penetration, or fisting. The frequency with which participants sought health care services was also assessed.

We provided clear and accessible definitions for uncommon or potentially unfamiliar terms such as “Cruising” [31], “Double Penetration” (DP) [9,19], “Chemsex” [29], and “Gouinage” [15,32] to ensure participants’ understanding. For the term “Chemsex,” participants were asked if they had consumed illicit drugs immediately before and during sexual intercourse within the past 12 months. Those who responded affirmatively were presented with a multiple-choice list to indicate specific drugs consumed [29].

The practices were defined based on previous studies [33-35], that are (1) DP: simultaneous sexual penetration by two or more penises. (2) Fisting or footing: anal penetration using the fist or foot. (3) Cruising: free, consensual, and anonymous sex practiced between men in public spaces such as parks, forests, beaches, or parking lots. (4) Gouinage: sexual activity without penetration.

### Data Analysis

Statistical analyses were performed using IBM SPSS 27.0 software (SPSS Inc). An initial exploratory analysis described the distribution of predictor variables across the study’s age groups of interest (<25 y, 25‐49 y, and ≥50 y). Data were presented in absolute frequencies, and percentage frequencies were calculated based on column totals, with denominators representing the subsamples from each age group. Associations between predictor variables and age groups were assessed using Pearson *χ*^2^ test with the Monte Carlo permutation method (999 simulations and a 95% confidence level), with a significance level of 5% (*P* value <.05).

Bivariate analyses were then conducted for each age group. Results were expressed in absolute frequencies, with percentage frequencies calculated based on row totals of the contingency table, using denominators representing the fraction of exposed and nonexposed categories for each predictor variable. Variables for inclusion in the multivariate model were selected using Pearson *χ*^2^ test with the Monte Carlo permutation method (999 simulations and a 95% confidence level), applying a statistical criterion of *P* value <.20 at this stage. Crude prevalence ratios (PRs) with their respective 95% CIs were calculated to measure the strength of association between the outcome (intention to use PrEP among MSM aged <25 y, 25‐49 y, and ≥50 y) and predictor variables, including social characteristics, sexual partnerships, forms of HIV prevention adopted, reasons for not using condoms in recent relationships, sexual practices, STI history, and willingness to use PrEP, given that the prevalence of PrEP intention exceeded 10%.

The final stage involved multivariate modeling to identify factors independently associated with the intention to use PrEP within each age group. We used the generalized linear model with a Poisson distribution and a log-linear link function. Adherence to the Poisson distribution was tested using the Kolmogorov-Smirnov test (*P* value >.05). In additionally, the equidispersion assumption was evaluated by comparing the variance and the mean of each outcome.

To compute adjusted prevalence ratios (aPRs) and their corresponding 95% CI, we used a hybrid parameter estimation method with a robust variance estimator and Type III analysis to test model effects. The omnibus test was used to evaluate whether the final multivariate model provided a better explanation of the prevalence of PrEP intention compared to a model that only included the intercept, with a significance level of 5% (*P* value <.05). The Akaike Information Criterion, deviance, and log-likelihood parameters served as criteria for selecting the best-fitting model, with lower values indicating better fit. The significance of aPRs for variables in the final model was determined using the Wald *χ*^2^ test, with variables having a *P* value <.05 considered significant.

### Ethical Considerations

The project obtained approval from the Ethics Committee of the Ribeirão Preto School of Nursing, Brazil, under opinion number 4,163,084. An online Informed Consent Form ( was provided and the platform used to host the collected data allowed for anonymization of all participants. All participants provided informed consent electronically before initiating the survey.

The online data collection platform ensured the anonymity of participants through mechanisms that prevented the identification of IP addresses and other traceable information. No identifying personal data were collected. Responses were de-identified at the source, and all data were stored on secure, password-protected servers with restricted access.

Following the completion of the study, participants received HIV prevention information and were directed to institutional websites for additional details on HIV/AIDS prevention. For individuals interested in PrEP usage who provided their contact email, a list of centers offering PrEP consultations in their state/region was supplied.

No monetary or material compensation was offered to participants. Participation was entirely voluntary and unpaid.

## Results

We recorded 8620 accesses to the survey form, of which 4341 responses or participants met the study’s inclusion criteria. Table 1 presents the baseline characteristics of the participants, who were predominantly cisgender (4216/4341, 97.1%), with ≥12 years of education (3298/4341, 76.0%), and native to Brazil (3686/4341; 84.9%). The majority identified as homosexual (3553/4341; 81.8%), were single (3000/4341; 69.1%), and reported having both fixed and casual partners (4010/4341; 92.4%). In addition, most had at least one casual sexual partner in the last 30 days (3838/4341; 88.4%) and reported meeting 1 to 3 casual sexual partners through apps in the past 6 months (2018/4341; 46.5%).

**Table 1.** Bivariate analysis of social, demographic and behavioral factors associated with intention to use PrEP among MSM by age group, a cross-sectional study, 2020‐2021, Brazil.

Variables	Age group																	
	<25 years (n=1702)						25‐49 years (n=2238)						≥50 years (n=401)					
	Intention to use PrEP																	
	Yes (n=925)		No (n=777)		PR (95% CI)	*P* value	Yes (n=1233)		No (n=1005)		PR (95% CI)	*P* value	Yes (n=232), n (%)		No (n=169), n (%)		PR (95% CI)	*P* value
	n (%)		n (%)				n (%)		n (%)				n (%)		n (%)			
Social characteristics																		
Gender identity						.07^a^						.15^a^						.66
Cisgender [ref]	911 (54.3)		766 (45.7)		—		1186 (55.2)		961 (44.8)		—		227 (57.9)		165 (42.1)		—	
Transgender	9 (81.8)		2 (18.2)		1.51 (1.14‐2.00)		25 (62.5)		15 (37.5)		1.13 (0.89‐1.44)		4 (66.7)		2 (33.3)		1.15 (0.65‐2.04)	
Other	5 (35.7)		9 (64.3)		0.66 (0.33‐1.33)		22 (43.1)		29 (56.9)		0.78 (0.57‐1.07)		1 (33.3)		2 (66.7)		0.58 (0.12‐2.86)	
Schooling						.39						.04^a^						.33
< 12 years [ref]	278 (56.0)		218 (44.0)		0.96 (0.87‐1.05)		250 (59.7)		169 (40.3)		0.91 (0.83‐0.99)		79 (61.7)		49 (38.3)		0.91 (0.76‐1.08)	
≥ 12 years	647 (53.6)		559 (46.4)				983 (54.0)		836 (46.0)				153 (56.0)		120 (44.0)			
Immigrant						.20						.008^a^						.13
Yes [ref]	178 (51.3)		169 (48.7)		1.07 (0.96‐1.20)		44 (42.3)		60 (57.7)		1.32 (1.05‐1.65)		126 (61.8)		78 (38.2)		0.87 (0.74‐1.03)	
No	747 (55.1)		608 (44.9)				1189 (55.7)		945 (44.3)				106 (53.8)		91 (46.2)			
Partnerships																		
Are you attracted to women?						.25						.96						.42
Yes	152 (51.4)		144 (48.6)		0.93 (0.83‐1.05)		235 (55.3)		190 (44.7)		1.00 (0.91‐1.09)		42 (62.7)		25 (37.3)		1.10 (0.90‐1.35)	
No [ref]	773 (55.0)		633 (45.0)				998 (55.0)		815 (45.0)				190 (56.9)		144 (43.1)			
Type of relationship						.17^a^						.02^a^						.32
Single	614 (53.0)		544 (47.0)		—		834 (53.3)		732 (46.7)		0.89 (0.82‐0.96)		153 (55.4)		123 (44.6)		0.87 (0.73‐1.03)	
Steady [ref]	278 (57.9)		202 (42.1)		0.91 (0.83‐1.01)		352 (60.2)		233 (39.8)		—		71 (64.0)		40 (36.0)		—	
Polyamorous	33 (51.6)		31 (48.4)		0.89 (0.69‐1.14)		47 (54.0)		40 (46.0)		0.90 (0.73‐1.10)		8 (57.1)		6 (42.9)		0.89 (0.56‐1.44)	
Type of sexual partnership						<.001^a^						.02^a^						>.99
Steady [ref]	96	67.6	46	32.4	0.79 (0.69‐0.89)		104	64.2	58	35.8	0.85 (0.75‐0.96)		16	59.3	11	40.7	0.97 (0.70‐1.35)	
Casual	829	53.1	731	46.9			1129	54.4	947	45.6			216	57.8	158	42.2		
Preferred sexual position						.03^a^						.01^a^						.17^a^
Only oral [ref]	38	43.7	49	56.3	-		59	42.8	79	57.2	-		10	40.0	15	60.0	-	
Insertive (top)	90	49.7	91	50.3	1.14 (0.86‐1.51)		132	53.7	114	46.3	1.25 (1.00‐1.57)		20	50.0	20	50.0	1.25 (0.71‐2.21)	
Receptive (bottom)	348	58.0	252	42.0	1.33 (1.04‐1.70)		447	57.4	332	42.6	1.34 (1.10‐1.64)		71	59.2	49	40.8	1.48 (0.89‐2.45)	
Versatile	449	53.8	385	46.2	1.23 (0.96‐1.58)		595	55.3	480	44.7	1.29 (1.06‐1.58)		131	60.6	85	39.4	1.52 (0.93‐2.48)	
Declare HIV sorology status in the apps						.01^a^						.22						.18^a^
Yes	77	45.6	92	54.4	0.82 (0.65‐1.04)		162	58.7	114	41.3	1.07 (0.97‐1.20)		75	63.0	44	37.0	1.13 (0.95‐1.35)	
No [ref]	848	55.3	685	44.7			1071	54.6	891	55.4			157	55.7	125	44.3		
Number of partners in the last 30 days						.01^a^						.56						.17
None [ref]	145	62.8	86	37.2	-		140	58.3	100	41.7	-		23	71.9	9	28.1	-	
1	391	54.0	333	46.0	0.86 (0.76‐0.97)		541	54.5	451	45.5	0.93 (0.83‐1.05)		65	53.7	56	46.3	0.75 (0.57‐0.98)	
≥2	389	52.1	358	47.9	0.83 (0.73‐0.94)		552	54.9	454	45.1	0.94 (0.83‐1.06)		144	58.1	104	41.9	0.81 (0.63‐1.03)	
Number of partners from apps in the last 6 mo																		
None [ref]	162	56.8	123	43.2	-	.46	181	54.5	151	45.5	-	.83	37	56.1	29	43.9	-	.57
1‐3	453	54.8	374	55.2	0.96 (0.86‐1.09)		568	54.6	473	45.4	1.00 (0.89‐1.12)		92	61.3	58	38.7	1.09 (0.85‐1.40)	
≥4	310	52.5	280	47.5	0.92 (0.81‐1.05)		484	56.0	381	44.0	1.03 (0.91‐1.15)		103	55.7	82	44.3	0.99 (0.77‐1.27)	
Forms of HIV prevention adopted.																		
Sexual abstinence																		
Yes [ref]	78	67.2	38	32.8	0.79 (0.69‐0.91)	.01^a^	109	64.1	61	35.9	0.85 (0.75‐0.95)	.02^a^	16	66.7	8	33.3	0.86 (0.64‐1.15)	.40
No	847	53.4	739	46.6			1124	54.4	944	45.6			216	57.3	161	42.7		
To cum outside																		
Yes [ref]	305	63.1	178	36.9	0.81 (0.74‐0.88)	<.001^a^	394	59.8	265	40.2	0.89 (0.82‐0.96)	.004^a^	77	63.6	44	36.4	0.87 (0.73‐1.03)	.15
No	620	50.9	599	49.1			839	53.1	740	46.9			155	55.4	125	44.6		
Being top																		
Yes [ref]	23	45.1	28	54.9	1.21 (0.89‐1.65)	.18^a^	33	55.0	27	45.0	1.00 (0.79‐1.26)	>.99	6	66.7	3	33.3	0.87 (0.54‐1.38)	.74
No	902	54.6	749	45.4			1200	55.1	978	44.9			226	57.7	166	42.3		
Gouinage																		
Yes [ref]	8	100.0	-	-	0.54 (0.52‐0.57)	.01^a^	6	100.0	-	-	0.55 (0.53‐0.57)	.04^a^	2	66.7	1	33.3	0.87 (0.39‐1.94)	>.99
No	917	54.1	777	45.9			1227	55.0	1,005	45.0			230	57.8	168	42.2		
Condom use																		
Yes [ref]	83	59.3	57	40.7	0.91 (0.79‐1.05)	.22	116	54.2	98	45.8	1.02 (0.89‐1.16)	.83	14	51.9	13	48.1	1.12 (0.77‐1.63)	.55
No	842	53.9	720	46.1			1117	55.2	907	44.8			218	58.3	156	41.7		
Justification for not using condoms in recent relationships																		
Partner uses PrEP																		
Yes [ref]	78	67.2	38	32.8	0.79 (0.69‐0.91)	.01^a^	109	64.1	61	35.9	0.85 (0.75‐0.95)	.02^a^	16	66.7	8	33.3	0.86 (0.64‐1.15)	.40
No	847	53.4	739	46.6			1124	54.4	944	45.6			21	57.3	161	42.7		
Known/repeat partner																		
Yes [ref]	620	50.9	599	49.1	1.24 (1.08‐1.42)	<.001^a^	839	53.1	740	46.9	1.13 (1.04‐1.22)	.004^a^	155	55.4	125	44.6	1.15 (0.97‐1.36)	.15
No	305	63.1	178	36.9			394	59.8	265	40.2			77	63.6	44	36.4		
Good physical appearance																		
Yes [ref]	23	45.1	28	54.9	1.21 (0.89‐1.65)	.18^a^	33	55.0	27	45.0	1.00 (0.79‐1.26)	>.99	6	66.7	3	33.3	0.87 (0.54‐1.38)	.74
No	902	54.6	749	45.4			1200	55.1	978	44.9			226	57.7	166	42.3		
Partner claims not to have an STI																		
Yes [ref]	106	60.6	69	39.4	0.89 (0.72‐1.08)	.09^a^	125	59.0	87	41.0	0.93 (0.82‐1.05)	.25	23	62.2	14	37.8	0.92 (0.71‐1.21)	.61
No	819	53.6	708	46.4			1108	54.7	918	45.3			209	57.4	155	42.6		
Partner reports having recently been tested																		
Yes [ref]	830	53.9	711	46.1	1.10 (0.89‐1.35)	.21	1113	54.8	919	45.2	1.06 (0.94‐1.20)	.34	210	56.9	159	43.1	1.21 (0.94‐1.55)	.26
No	95	59.0	66	41.0			120	58.3	86	41.7			22	68.8	10	31.3		
Sex intensity																		
Yes	222	64.0	125	36.0	1.23 (1.06‐1.43)	<.001^a^	309	60.9	198	39.1	1.14 (1.05‐1.24)	.003^a^	61	64.9	33	35.1	1.17 (0.97‐1.39)	.12
No [ref]	703	51.9	652	48.1			924	53.4	807	46.6			171	55.7	136	44.3		
Sexually gifted partner																		
Yes	56	56.6	43	43.4	1.04 (0.80‐1.37)	.65	100	54.1	85	45.9	0.98 (0.85‐1.13)	.82	14	58.3	10	41.7	1.01 (0.71‐1.43)	>.99
No [ref]	869	54.2	734	45.8			1133	55.2	920	44.8			218	57.8	159	42.2		
Sexual practices																		
The last person you had sex with was HIV+																		
Yes	25	62.5	15	37.5	1.15 (0.90‐1.47)	.29	26	60.5	17	39.5	1.10 (0.86‐1.40)	.54	7	87.5	1	12.5	1.53 (1.16‐2.01)	.15
No [ref]	900	54.2	762	45.8			1207	55.0	988	45.0			225	57.3	168	42.7		
Group sex																		
Yes	327	42.3	446	57.7	0.66 (0.60‐0.72)	<.001^a^	458	48.5	486	51.5	0.81 (0.75‐0.88)	<.001^a^	81	46.8	92	53.2	0.71 (0.59‐0.85)	<.001
No [ref]	598	64.4	331	35.6			775	59.9	519	40.1			151	66.2	77	33.8		
Frequent barebacking																		
Yes	893	99.9	1	0.1	25.22 (17.73‐35.89)	<.001^a^	1233	100.0	-	-	-	<.001^a^	232	98.3	4	1.7	-	<.001
No [ref]	32	4.0	776	96.0			-	-	1,005	100.0			-	-	165	100.0		
Double penetration																		
Yes	262	71.2	106	28.8	1.43 (1.24‐1.65)	<.001^a^	320	66.1	164	33.9	1.27 (1.17‐1.37)	<.001	57	73.1	21	26.9	1.35 (1.14‐1.59)	.003
No [ref]	663	49.7	671	50.3			913	52.1	841	47.9			175	54.2	148	45.8		
Chemsex practice																		
Yes	270	58.3	193	41.7	1.10 (0.96‐1.28)	.05^a^	333	55.0	273	45.0	1.00 (0.92‐1.08)	.96	59	66.3	30	33.7	1.20 (1.00‐1.43)	.07
No [ref]	655	52.9	584	47.1			900	55.1	732	44.9			173	55.4	139	44.6		
Fisting/footing																		
Yes	85	57.8	62	42.2	1.07 (0.86‐1.34)	.38	100	52.4	91	47.6	0.95 (0.82‐1.09)	.45	19	59.4	13	40.6	1.03 (0.76‐1.39)	>.99
No [ref]	840	54.0	715	46.0			1133	55.3	914	44.7			213	57.7	156	42.3		
Cruising practice																		
Yes	18	64.3	10	35.7	1.19 (0.74‐1.89)	.28	20	62.5	12	37.5	1.14 (0.87‐1.49)	.48	-	-	-	-	-	-
No [ref]	907	54.2	767	45.8			1213	55.0	993	45.0			232	57.9	169	42.1		
STIs History																		
Had clamydia diagnosis																		
Yes	89	53.3	78	46.7	0.98 (0.79‐1.22)	.77	220	59.8	148	40.2	1.10 (1.00‐1.21)	.05^a^	25	52.1	23	47.9	0.89 (0.67‐1.18)	.44
No [ref]	836	54.5	699	45.5			1013	54.2	857	45.8			207	58.6	146	41.4		
Had gonorrhea diagnosis																		
Yes	103	54.2	87	45.8	1.00 (0.81‐1.22)	.96	223	59.8	150	40.2	1.10 (1.00‐1.21)	.05^a^	28	50.9	27	49.1	0.86 (0.66‐1.14)	.30
No [ref]	822	54.4	690	45.6			1010	54.2	855	45.8			204	59.0	142	41.0		
Had syphilis diagnosis																		
Yes	125	51.0	120	49.0	0.93 (0.77‐1.12)	.25	258	60.1	171	39.9	1.12 (1.02‐1.22)	.02^a^	38	62.3	23	37.7	1.09 (0.88‐1.35)	.48
No [ref]	800	54.9	657	45.1			975	53.9	834	46.1			194	57.1	146	42.9		

a variables that met the statistical criterion of a *P* value <.20.

In terms of sexual practices, nearly half of the sample identified as versatile (2125/4341; 49.0%) and reported engaging in group sex (1890/4341; 43.5%). Furthermore, the majority indicated frequent bareback sex (2363/4341; 54.4%). Higher-risk HIV exposure practices, such as DP (930/4341; 21.4%) and chemsex (1,158/4341; 26.7%), were reported by approximately one-quarter of the respondents.

Regarding HIV/AIDS prevention methods, most MSM reported widespread use of less effective practices such as interrupted intercourse (3078/4341; 70.9%), insertive sexual practices (4324/4341; 99.6%), gouinage (4324/4341; 99.6%), and sexual abstinence (4031/4341; 92.9%). Condom use during at least one of the most recent sexual encounters was reported by 91.2% (3960/4341) of participants. Among the reasons for not using condoms during sexual encounters, participants cited not using them with known sexual partners (3078/4341; 70.9%) and with partners who had recently tested for HIV (3,942/4341; 90.8%).

The majority of participants reported no previous history of chlamydia (3758/4341; 86.6%), gonorrhea (3723/4341; 85.8%), or syphilis (3606/4341; 83.1%), and frequently attended health care services (3120/4341; 71.9%).

PrEP intention was expressed by more than half of the participants (2390/4341; 55.1%). Bivariate analysis was conducted for each of the 3 age groups, and variables meeting the statistical criteria for inclusion in the multivariate modeling stage were selected accordingly (Table 1).

Table 2 presents the independent factors associated with the intention to use PrEP in each age group, along with their respective measures of association. A total of 5 variables were independently associated with the outcome of interest in the models for the age subgroups <25 and ≥50 years, while 9 variables were included in the final model for adults aged 25‐49 years.

**Table 2.** Multivariate analysis of factors associated with intention to use PrEP among MSM by age group, a cross-sectional study, 2020‐2021, Brazil.

Variables	*β*	Adjusted prevalence ratio	95% CI		*P* value
			Lower	Superior	
<25 years^a^					
Intercept	−2.99	0.05	0.03	0.07	<.001
Frequent bareback	3.23	25.40	18.18	35.48	<.01
Transgender identity	.03	1.03	1.01	1.05	.01
Casual sexual partnership	−.02	0.98	0.97	0.99	.01
Chemsex practice	−.04	0.96	0.94	0.98	<.01
≥1 sexual partner in the last 30 days	−.25	0.78	0.72	0.85	<.01
25‐49 years^b^					
Intercept	−.74	0.48	0.39	0.59	<.01
Adopt a receptive position	.24	1.27	1.04	1.55	.02
Being an immigrant	.23	1.26	1.02	1.56	.03
Be versatile	.21	1.23	1.01	1.49	.04
Double penetration practice	.20	1.22	1.13	1.32	<.01
Inconsistent condom use with casual partners	.13	1.14	1.05	1.24	.01
Belief that condoms can be abandoned due to age of sex	.12	1.13	1.04	1.23	.04
Previous history of syphilis	.11	1.12	1.03	1.22	.01
Being single	−.11	0.90	0.83	0.97	.07
Belief that condoms can be abandoned if the partner is on PrEP^c^	−.20	0.82	0.72	0.93	.01
Gouinage practice	−.67	0.51	0.46	0.57	<.01
≥50 years^d^					
Intercept	−.73	0.48	0.40	0.58	<.01
Double penetration practice	.27	1.31	1.20	1.43	<.01
Adopt a receptive position	.24	1.27	1.04	1.55	.02
Being an immigrant	.24	1.27	1.03	1.58	.03
Be versatile	.21	1.23	1.01	1.50	.04
Chemsex practice	−.18	0.83	0.76	0.92	<.01
Group sex practice	−.20	0.82	0.75	0.89	<.01

a Deviance: 201.32; Akaike Information Criterion: 2,067.32; BIC: 2,110.84; Omnibus test: *χ*2(7)=926.74; *P* value <.001.

b Deviance: 1,433.85; Akaike Information Criterion: 3,925.85; BIC: 4,000.12; Omnibus test: *χ*2(12)=36.21; *P* value <.001.

c PrEP: pre-exposure prophylaxis.

d Deviance: 1,435.809; Akaike Information Criterion: 3,917.81; BIC: 3,963.52; Omnibus test: *χ*2(7)=34.25; *P* value <.001).

The practice of chemsex was associated with a lower prevalence of the intention to use PrEP among MSM aged <25 years (aPR: 0.96; 95% CI 0.94‐0.98) and those aged ≥50 years (aPR 0.83; 95% CI 0.76‐0.92). Certain variables were shared among the final models of different subgroups. The practice of DP was associated with a higher prevalence of the intention to use PrEP among MSM aged 25‐49 years (aPR: 1.22; 95% CI 1.13‐1.32) and those aged ≥50 years (aPR: 1.31; 95% CI 1.20‐1.43). Being bottom or receptive (aPR: 1.27; 95% CI 1.04‐1.55) or versatile (aPR 1.23; 95% CI 1.01‐1.50) was also associated with a higher prevalence of PrEP intention among MSM aged 25‐49 years and ≥50 years.

Frequent bareback sex was associated with a prevalence 25.4 times higher for the intention to use PrEP among MSM aged <25 years. Conversely, having one or more casual sexual partners in the last 30 days was associated with a 22% lower frequency of PrEP intention.

Among MSM aged 25‐49 years, the prevalence of intention to use PrEP was over 10% (258/429) higher among those with specific characteristics, such as a previous diagnosis of syphilis (aPR 1.12; 95% CI 1.03‐1.22) and nonuse of condoms due to the intensity of sex (aPR 1.13; 95% CI 1.04‐1.23) or when sexual partners were known (aPR: 1.14; 95% CI 1.05‐1.24). However, being single (aPR: 0.90; 95% CI 0.83‐0.97), having a sexual partner who used PrEP (aPR: 0.82; 95% CI 0.72‐0.93), and using gouinage as an HIV/AIDS prevention practice were exclusively associated with lower prevalences of PrEP intention.

The practice of group sex was independently associated with a lower prevalence of PrEP intention exclusively among MSM aged ≥50 years (aPR: 0.82; 95% CI 0.75‐0.89).

## Discussion

### Principal Findings

This study, which explores factors associated with the intention to use PrEP among MSM across various age groups, found that more than half of the participants expressed an intention to adopt PrEP. However, this intention varies significantly between age groups, emphasizing distinct behaviors and perceptions that may influence the decision to use PrEP. Gaining an understanding of these age-specific nuances can aid in the development and implementation of tailored strategies to enhance PrEP awareness and adherence within this population.

In examining the factors that influence the intention to use PrEP among different age groups, our findings reveal that this intention is significantly shaped by sexual practices, risk perception, and sociodemographic characteristics. While some factors are shared across age groups, recognizing the unique characteristics and needs of each age category provides a more comprehensive understanding of the topic and highlights opportunities for targeted interventions.

### Intention to Use PrEP Among Young Individuals (Under 25 Years)

In the age group under 25 years, the practice of unprotected sex or frequent barebacking was associated with a substantial increase in the intention to use PrEP, with a prevalence 25.4 times higher. This finding is crucial for discussions on the need for targeted interventions aimed at young individuals engaging in higher-risk behaviors for HIV infection. A 2021 study analyzing the influence of consuming bareback-type sexually explicit media among Brazilian MSM found that a preference for condomless scenes, casual sexual partnerships, and multiple partners was linked to an increased likelihood of engaging in condomless anal sex, particularly among younger MSM populations [36].

Another study conducted in Brazil demonstrated that MSM aged 15‐19 who engage in barebacking had a higher likelihood of initiating PrEP use [37]. However, data also show that in Brazil, MSM aged 18‐24 years are twice as likely to discontinue PrEP treatment after the first month compared to older MSM. Risky behaviors, transactional sex (in exchange for gifts or money), a lower perception of HIV risk, and illicit drug use further contribute to a higher prevalence of HIV infection within this group [38].

A study involving adolescent MSM in 3 Brazilian capitals reported a 79.9% prevalence of barebacking within the past 6 months. Another noteworthy finding indicated that being transgender was associated with a higher intention to use PrEP, potentially due to the unique risk factors this population faces throughout their lives [39].

Similarly, in our study, the practice of chemsex among individuals under 25 years was associated with a lower prevalence of PrEP intention, as was having multiple casual sexual partners in the past 30 days. This suggests complex behavioral nuances within this subgroup. One possible interpretation is that young individuals involved in chemsex may significantly underestimate their HIV exposure risk [40]. This underestimation could stem from the effects of substances consumed during chemsex, which may impair judgment, alter risk perception, and provide a temporary but false sense of invulnerability [41].

The prevalence of chemsex among young individuals may reflect sociocultural, moral, and health care dynamics linked to the pursuit of pleasure characteristic of this age group. This behavior often involves the discovery of the body and intensified emotional connections during social events or parties [42-44]. Such a focus on immediate gratification can overshadow long-term health considerations, making HIV prevention efforts seem less pressing or relevant [45,46]. Early exposure to social networks and the early initiation of sexual activity further increase the risks of engaging in chemsex and partially adhering to PrEP [47].

In addition, substance use associated with chemsex may have psychological effects, such as depression or anxiety, which influence decision-making. These mental health conditions can decrease motivation to seek and adhere to HIV prevention strategies like PrEP [48].

Thus, sociocultural factors, including limited awareness of PrEP, internalized stigma due to negative attitudes toward homosexuality and sexual activity, and the stigmatization of PrEP users, emerge as critical barriers to adopting this preventive measure.

### Intention to Use PrEP Among Adults (Intermediate Age Range of 25 to 49 Years)

For the intermediate adult age group, 9 variables showed significant associations. In this group, “personal experiences with STIs” appear to serve as a “wake-up call” for the risk of contracting HIV, increasing awareness of the risk and encouraging the pursuit of combined or additional prevention measures, such as PrEP. Unlike what was observed in younger MSM, past experiences in this group directly influence risk perception and the decision to adopt preventive measures, highlighting how accumulated experiences over time can be a critical factor for awareness and adherence to PrEP [49]. This result aligns with existing literature, which emphasizes that older age can be significantly associated with increased PrEP use among MSM [10,16,49].

PrEP intention is positively impacted by the role of sexual position, with individuals assuming insertive, versatile, or DP roles being more likely to adopt PrEP, while those engaging in nonpenetrative practices (such as gouinage) are less inclined to do so [50,51]. This finding may reflect an awareness that certain sexual positions carry higher HIV transmission risks due to increased exposure to the virus [52]. However, it is essential to interpret these results cautiously, as any unprotected sexual exposure carries inherent risks. Furthermore, practices such as DP elevate transmission risk due to the challenging nature of the practice, which can increase the likelihood of microlesions and, consequently, vulnerability to infections [34,53].

Situational factors, such as the intensity of sexual practices and trust in partners who claim to be using PrEP, emerged as significant contributors to the likelihood of PrEP adherence. These factors underscore the multifactorial nature of motivations for PrEP use among MSM in this age range, highlighting the importance of clinical consultations that adopt a holistic approach, seeing MSM comprehensively. It is crucial for these consultations to consider the complexity of individual experiences and risk perceptions to understand and effectively implement PrEP within the context of MSM’s lives.

An interesting finding in this group pertains to the context of migration, where immigrant MSM were more likely to adhere to PrEP. MSM immigrants may face challenges such as limited health care access, stigma, and conditions that heighten their vulnerability, making them more inclined to seek additional protective measures like PrEP as a form of individual health control [20,54].

This underscores the need for prevention and education campaigns about PrEP to encompass a broad spectrum of life situations and risk perceptions. These variations highlight the urgent need for personalized and adaptable prevention strategies that consider the unique characteristics and needs of each age subgroup [55,56]. This context emphasizes the complexity of decisions regarding PrEP usage and reinforces the significance of culturally sensitive public health approaches informed by the diverse realities experienced within the MSM community.

### Intention to Use PrEP Among Older MSM (Age Range Above 50 Years)

Limited attention has been given to analyzing the factors related to PrEP use among older MSM, and the findings from our study imply a range of implications and underlying dynamics that require careful examination [10,16,57]. Social stigma surrounding HIV/AIDS, combined with the marginalization of non-conventional sexual behaviors, can lead to reluctance or hesitation in seeking information and prevention services, including HIV testing and counseling. Older individuals may feel uncomfortable discussing their sexual practices [10,16].

As MSM age, changes in sexual practices, social networks, and risk perception often occur, which can impact their decision to initiate PrEP [16]. In this age group, there is an increasing convergence of factors with younger adults, particularly regarding the adoption of sexual positions (passive and versatile individuals are more likely to adopt PrEP) and engagement in high-risk sexual activities. This finding is supported by a multicenter study conducted with older Portuguese-speaking MSM, which showed that those who adopted a receptive or versatile sexual position were more likely to use PrEP [16]. The study emphasizes that HIV exposure among older MSM is influenced not only by age or isolated behavioral factors but also by an accumulated perception of risk and willingness to adopt protective measures.

In this age group, it is noteworthy that chemsex and group sex practices were associated with a lower intention to use PrEP, a finding that contrasts with trends observed in younger counterparts [19,58]. One possible explanation is that among older MSM, chemsex tends to occur less frequently and in more situational contexts, often being perceived as an isolated event rather than a routine sexual behavior. This occasional nature of their practices may lead them to underestimate their risk, viewing PrEP as an unnecessary or excessive measure for situations they perceive as exceptional. Unlike younger individuals, who often see chemsex as part of a continuous high-risk practice associated with parties and sexual encounters, and thus opt for PrEP to mitigate frequent exposure, older MSM may not perceive themselves as being in a constant risk context that would justify regular preventive use [16,19,59].

Additionally, risk perception among older MSM is often shaped by traditional or conservative views on sexuality and self-image [17], leading them to perceive PrEP as a strategy targeted at younger or more sexually “promiscuous” populations. This internalized stigma, which associates PrEP use with behaviors deemed morally transgressive, can discourage interest in the prophylaxis. For some, adherence to PrEP may seem unnecessary or even damaging to their identity, revealing a significant barrier related to self-image and the influence of social norms on the use of preventive strategies [16,17].

Another relevant factor is what can be described as “prevention fatigue” among older MSM. After decades of exposure to the HIV/AIDS epidemic and adherence to prevention campaigns primarily focused on condom use and reducing risky practices, many may feel exhausted by the constant vigilance required for prevention. This fatigue can lead to apathy or resignation toward new preventive interventions, such as PrEP, resulting in passive resistance to its adoption. This fatigue is particularly complex, as it reflects not only practical exhaustion but also emotional strain from prolonged exposure to stigma and fear surrounding HIV.

Additionally, it is important to consider that older MSM may experience social isolation, lower health literacy, and limited access to support networks or groups that discuss sexual health. This can hinder their access to updated information on PrEP and other preventive interventions. Such isolation, often exacerbated by age-related stigma and barriers within health care settings, contributes to a lack of awareness and misinformation about PrEP, reinforcing perceptions that it is unnecessary or unsuitable for this age group [16,17].

These findings underscore the need for personalized prevention approaches that consider the specific contexts and values of MSM over the age of 50. Interventions targeting this population should adopt inclusive language that is sensitive to age-related issues, emphasizing the benefits of PrEP without associating it exclusively with a specific age group or lifestyle.

### Implications for Public Health and Surveillance

Our findings carry critical and urgent implications for public health and surveillance. Public health strategies must consider generational differences in attitudes, knowledge, and behaviors related to PrEP and HIV. Educational campaigns and awareness programs should be tailored to address the unique and specific needs of each age group, using the most effective language and communication channels for each, rather than using a single, generic approach.

Furthermore, public health initiatives should work to expand access to PrEP by addressing specific barriers faced by different generations. This includes reducing stigma surrounding PrEP use, ensuring financial and physical accessibility, and providing targeted education to healthcare providers on how to effectively address issues of sexuality and HIV prevention with MSM of all ages.

Our conclusions also underscore the importance of integrating sexual and mental health services, particularly to address issues such as stigma, substance use, and social isolation. Integrated services can better address the complex health needs of MSM, promoting a holistic and effective approach to HIV prevention.

Moreover, public health must continue to monitor trends in PrEP use and HIV infection rates across different generational groups of MSM. This surveillance can help identify gaps in prevention and treatment efforts, informing the development of targeted interventions. In addition, research should investigate the underlying reasons for generational differences in PrEP intention to inform more effective intervention strategies.

It is also important to consider the variability of socialization patterns and affective and sexual interactions, sociocultural transformations, and the influence of sexually explicit media content. The appeal of engaging in high-risk sexual practices, such as chemsex and DP—practices highlighted in our study—can lead to greater involvement in group sexual activities, increasing the risk of sexually transmitted infections, especially when there is limited knowledge of individual serological status and preventive measures taken by each participant [9,60,61].

In addition, the study of health behaviors among adult and elderly men, who are often overlooked in health priority agendas, must not be neglected. Research has highlighted the consequences of this oversight, indicating that older men often have insufficient information about HIV/AIDS compared to younger men, a gap that is reflected in their perceptions of individual health, sexual activity, and HIV testing behaviors.

### Limitations

Our study presents several limitations that warrant discussion. First, the data collection method may introduce significant information biases. While the use of an online survey and a “snowball” sampling strategy adapted to the virtual environment is both innovative and practical, it may not capture a fully representative sample of the Brazilian MSM population. This approach tends to include individuals with greater internet access and technological familiarity, potentially excluding those from less urbanized regions or with limited digital access—a challenge that is especially pronounced in a vast country like Brazil. Moreover, our initial recruitment through 2 dating apps may have skewed the sample toward individuals who are more sexually active or seeking casual relationships, potentially not reflecting the broader practices and intentions regarding PrEP use among all MSM.

Additionally, although the study addresses key sociodemographic and behavioral variables, it may not fully encompass the complex sociostructural contexts that influence PrEP utilization intentions. Critical factors such as social stigma, discrimination, social support, mental health, and access to health care services play significant roles in individual health decisions and may vary widely among different MSM subgroups. Moreover, cultural and media narratives, particularly concerning chemsex and other sexual practices, may have distinct impacts across different age groups, which were not fully explored in this study.

### Conclusions

The adoption of HIV preventive measures, such as PrEP, among Brazilian MSM reveals complex and varied nuances across different age groups. Our findings indicate that more than half of the participants expressed an intention to adopt PrEP; however, this intention varies significantly among different age groups, reflecting distinct behaviors and perceptions that influence PrEP use decisions.

For individuals under 25 years of age, high-risk practices such as frequent bareback sex were strongly associated with a greater intention to use PrEP, potentially reflecting a heightened perception of HIV risk or a response to their vulnerability. In contrast, having multiple casual sexual partners in the last 30 days was associated with a lower likelihood of intending to use PrEP, suggesting potential challenges in risk perception or PrEP accessibility for these individuals.

Among those in the intermediate age range (25 to 49 years), factors such as engaging in DP, being passive or versatile, and having a previous diagnosis of syphilis were associated with a higher intention to use PrEP. For MSM aged 50 years or older, DP and receptive or versatile sexual positions were linked to a greater intention to use PrEP, while group sex was negatively associated. These differences underscore the importance of personalized PrEP promotion approaches that consider the diverse behaviors, attitudes, and challenges faced by MSM across different ages. Understanding these nuances is essential for developing more effective public health strategies aimed at increasing PrEP awareness and adherence, ultimately contributing to a reduction in HIV infection rates among Brazilian MSM.

## Abbreviations

aPR: adjusted prevalence ratio
DP: double penetration
PR: prevalence ratio
PrEP: Pre-Exposure Prophylaxis
STIs: sexually transmitted infections
WHO: World Health Organization
